# Speeding-up the Determination of Protein–Ligand Affinities by STD NMR: The Reduced Data Set STD NMR Approach (rd-STD NMR)

**DOI:** 10.1021/acs.analchem.3c03980

**Published:** 2024-01-02

**Authors:** Gabriel Rocha, Jonathan Ramírez-Cárdenas, M. Carmen Padilla-Pérez, Samuel Walpole, Ridvan Nepravishta, M. Isabel García-Moreno, Elena M. Sánchez-Fernández, Carmen Ortiz Mellet, Jesús Angulo, Juan C. Muñoz-García

**Affiliations:** † Institute for Chemical Research (IIQ), CSIC—University of Seville, 49 Américo Vespucio St, 41092 Seville, Spain; ‡ Department of Organic Chemistry, Faculty of Chemistry, University of Seville, 1 Prof. García González St, 41012 Seville, Spain; § School of Pharmacy, University of East Anglia, Norwich Research Park, NR4 7TJ Norwich, United Kingdom; ∥ Cancer Research Horizons, The Beatson Institute, Garscube Estate, Switchback Road, Bearsden, Glasgow G61 1BD, United Kingdom

## Abstract

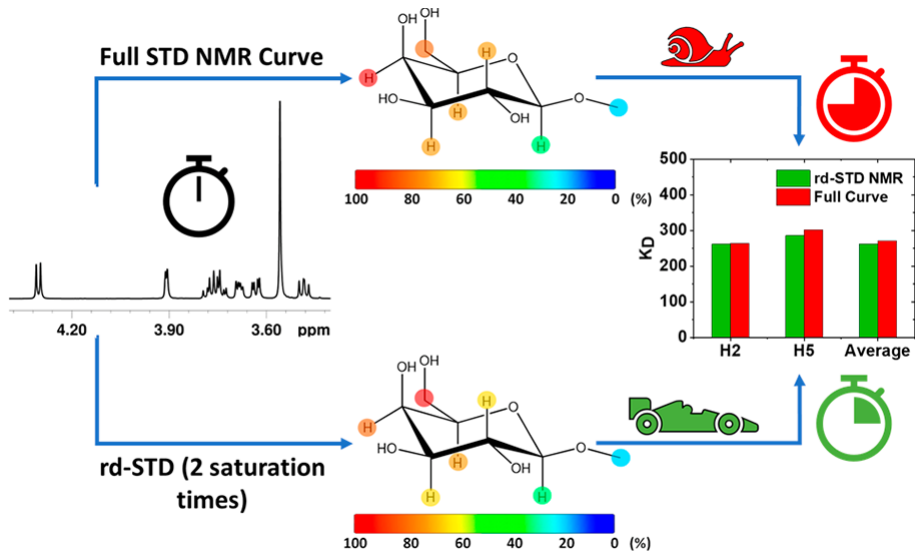

STD NMR spectroscopy is a powerful ligand-observed NMR tool for screening and characterizing the interactions of small molecules and low molecular weight fragments with a given macromolecule, identifying the main intermolecular contacts in the bound state. It is also a powerful analytical technique for the accurate determination of protein–ligand dissociation constants (*K*_D_) of medium-to-weak affinity, of interest in the pharmaceutical industry. However, accurate *K*_D_ determination and epitope mapping requires a long series of experiments at increasing saturation times to carry out a full analysis using the so-called STD NMR build-up curve approach and apply the “initial slopes approximation”. Here, we have developed a new protocol to bypass this important limitation, which allows us to obtain initial slopes by using just two saturation times and, hence, to very quickly determine precise protein–ligand dissociation constants by STD NMR.

NMR has become a powerful tool to monitor intermolecular interactions and characterize the structural and dynamic features of recognition processes at different levels of complexity.^1,2^ A significant number of NMR approaches have been developed and applied with remarkable success to different systems, particularly those involving receptor–ligand interactions of biological interest. Saturation transfer difference NMR (STD NMR) spectroscopy^3−5^ is one of the most powerful and versatile NMR techniques used to accurately determine affinities and analyze binding epitope mappings, especially in the case of weak protein–ligand interactions, having increasing application in both academic research and the pharmaceutical industry (e.g., for fragment-based drug discovery, FBDD).

At the molecular level, the biological activity of drugs on macromolecular receptors (usually proteins) is determined by the structural and energetic characteristics governing the formation of the receptor–ligand complex. Therefore, there is great interest in the pharmaceutical industry in new approaches to accurately and rapidly determine the typically weak affinities of protein fragments (hits) for FBDD. The acquisition of full STD build-up curves is essential to obtaining accurate binding epitopes and dissociation constants.^6^ However, in those cases where full STD NMR build-up analysis cannot be carried out (e.g., limitations in protein availability and/or stability), a single saturation time titration experiment will provide an upper limit of the dissociation constant.^7^ It is also worth noting that, for small molecule screening purposes, it is generally sufficient to perform single saturation time titration experiments since, normally, the ranking of compounds is more relevant at this stage than the accurate detemination of thermodynamic constants.

STD NMR intensities are proportional to the fraction of bound ligands in solution,^8^ but it is the monitoring of parameters proportional to the fraction of bound protein in titration experiments which can be used to determine target-ligand binding affinities. Mayer and Meyer proposed a conversion of STD intensities to amplification factors (STD-AF) by multiplying the observed STD by the molar excess of ligand over protein (ε):^4^
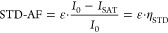
1As we previously demonstrated, differences in ^1^H T_1_ relaxation time constants at long saturation times along with fast rebinding effects within the relaxation time scale can have a strong impact on the determination of affinities.^6^ To remove the effects of these factors, ligand binding epitopes and protein–ligand dissociation constants (*K*_D_) have to be determined by monitoring STD NMR initial slopes (STD_0_)^6,9,10^ obtained from full STD build-up curves (STD initial growth rates). In this way, following the amplification factors determined from initial slopes (STD-AF_0_) over a series of titrations, it is possible to construct an isotherm that can be mathematically fit to the law of mass action equation^11^
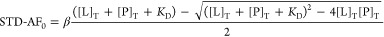
2where STD-AF_0_ is the STD initial slope (STD_0_) multiplied by the molar excess of ligand over protein (ε). Eq 2 yields both the equilibrium dissociation constant, *K*_D_, and the proportionality parameter β.

One of the well-known main drawbacks of employing the initial slope approximation for *K*_D_ determination is the typically long experimental times required. This can become an insurmountable problem for samples prone to degradation with time. To get accurate initial slopes from a nonlinear saturating hyperbolic STD build-up curve, typically six to nine data points are required to properly sample both the initial growth (e.g., starting at 0.5, 0.75, and 1 s) as well as the plateau (up to 6 to 8 s) of the curve. In addition, the full build-up curve has to be acquired for at least five to six ligand concentrations constituting the different abscissa data points in the binding isotherm. Thus, for instance, for an isotherm of six ligand concentrations and six saturation times for each build-up curve, the *K*_D_ determination by STD NMR can take roughly 2 to 3 days (note that, for the smallest ligand concentrations and saturation times, more scans and, hence, experimental time is required to obtain an interpretable spectra).

As STD NMR signals arise from intermolecular (protein–ligand) NOEs, we found inspiration in previous NOE data analysis that demonstrated a simplification of the analytical procedure by expanding the linear range of NOE build-up from 1D, 2D NOESY, and exchange experiments.^12,13^ Further, it is well established that transverse relaxation rates (R2) can be calculated with good accuracy by applying linearization and using two data points only instead of acquiring 16 to 32 data points to fit the corresponding exponential equation.^14^

In this work, we propose a new analytical approach, the reduced data set STD NMR (rd-STD NMR), to significantly reduce the large experimental time (e.g., 2 to 3 days) required for *K*_D_ determination by STD NMR (e.g., to 12 to 24 h). Our approach allows a savings of 60 to 80% of NMR experimental time and associated costs. Recently, Monaco et al. reported a new STD NMR method for the determination of protein–ligand dissociation constants based on chemical shift imaging and a concentration gradient of the small molecule.^7^ This imaging STD NMR protocol has the advantage of requiring one sample only and reducing the experimental time by approximately 80%. However, setting up the imaging STD NMR method requires more sophisticated sample preparation (i.e., the density of protein and ligand buffers have to be optimized, and the diffusion coefficient of the ligand must be determined) and data analysis (nonstandard spectra processing is required), which may seem tedious for standard NMR users. Further, high throughput analysis of tens to hundreds of fragment molecules would require significant steps of optimization. On the contrary, the rd-STD NMR protocol presented herein allows preservation of the ease of automatization and simplicity of sample preparation, STD NMR experimental acquisition, and spectra analysis, allowing also the savings of up to 80% of experimental time.

## Results and Discussion

### The Reduced-Data Set rd-STD NMR Approach: Apparent STD Initial Slopes from Linearized Analysis

Our new approach is based on the monoexponential description of the accumulation of the STD signal as a function of the saturation time:
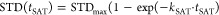
3where STD (*t*_SAT_) is the observed STD intensity at a given saturation time (*t*_SAT_), STD_MAX_ is the maximum STD intensity, and *k*_SAT_ is the saturation transfer constant. From eq 3, STD_MAX_ and *k*_SAT_ parameters are obtained by least-squares fitting of the experimental curve. The initial growth rate of the STD build-up curve (STD_0_) is defined as
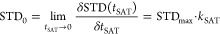
4By applying the Napierian logarithm (ln) transformation to eq 3, it can be rearranged as a linear equation:
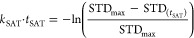
5where *k*_SAT_ is the slope of the straight line between two points (STD_MAX_ and STD_(*t*_SAT_)_) and STD_MAX_ can be approximated by measuring the STD intensity at a sufficiently long saturation time (STD_LONG_). Application of the two-point linearized form of the STD NMR initial slope approximation represented by eq 5 is what we call the reduced data set STD NMR approach (rd-STD NMR), as only two experimental data points are needed to determine the initial slope (STD_0_, eq 4; STD-AF_0_, eq 2). In this way, measuring a sufficiently long saturation time STD (STD_LONG_, assumed to be the asymptotic STD_MAX_ value) and a sufficiently short saturation time STD (STD_SHORT_, assumed to be at the growing section of the build-up curve), the apparent initial slope can be approached by
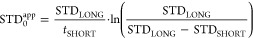
6Such an rd-STD NMR approximation constitutes a novel analytical protocol to accelerate the determination of ligand binding epitopes and protein–ligand dissociation constants. Here, we demonstrate, for five different protein–ligand complexes, that the rd-STD NMR approach can determine, in a much faster way than traditional STD NMR analysis, ligand binding epitopes and *K*_D_ values that are highly similar to those obtained by the traditional full curve method (similar to calorimetry values).

### Validation of the rd-STD NMR Approach 1: Analysis of Single-Ligand Concentration Apparent Initial Slope Data (STD_0_^app^). Structural Information from Binding Epitope Mapping

**Figure 1:**
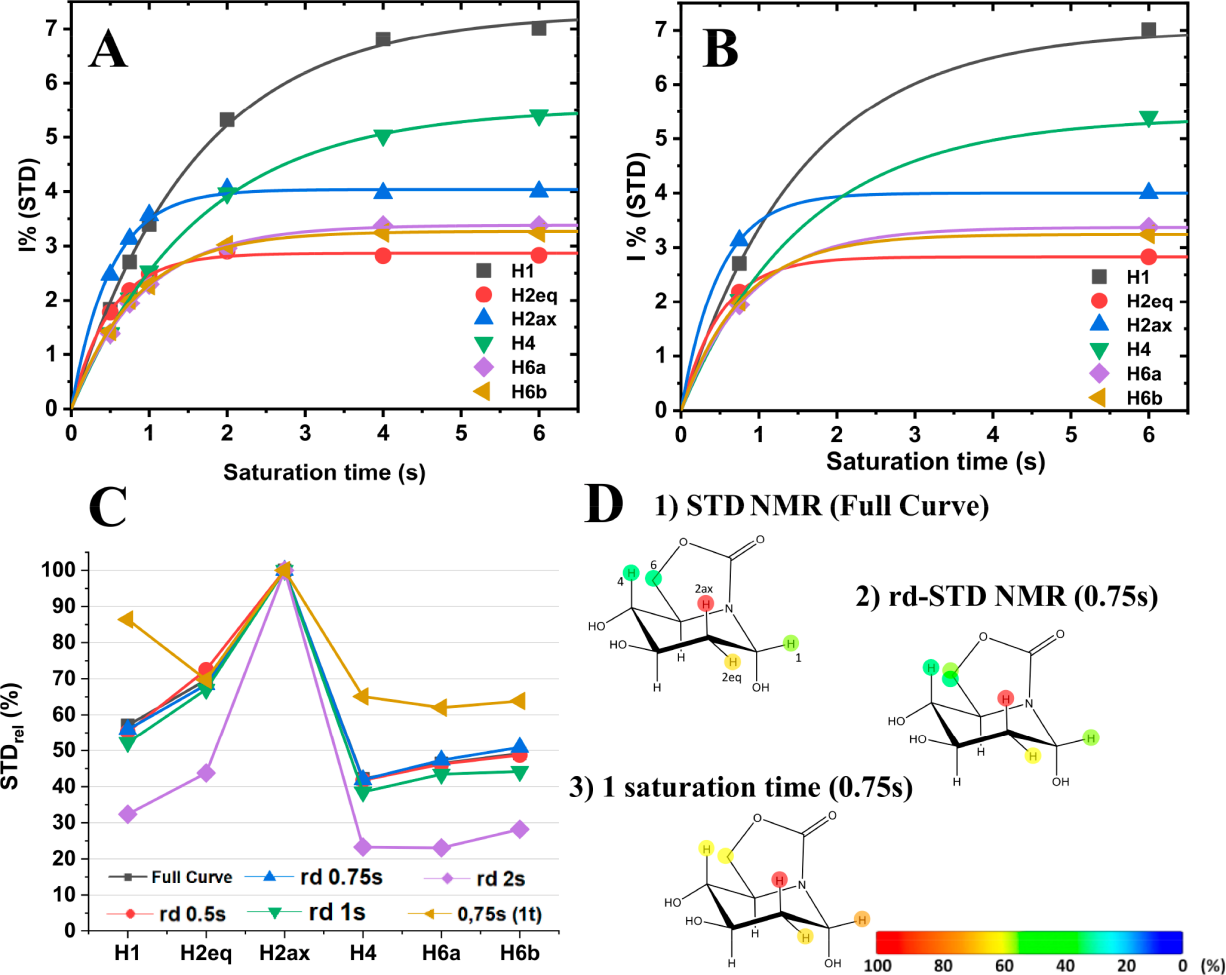
Mathematically fitted STD NMR build-up curve of ESF452 binding to α-glucosidase using (A) all saturation times and (B) the new rd-STD NMR approach using only 0.75 and 6 s as data points. (C) STD binding epitope mapping: STD relative values for each ESF452 proton obtained using the full build-up curve initial slopes method (black dots and line), the new rd-STD NMR approach with STD_SHORT_ of 0.5, 0.75, 1 and 2 s (red, blue, green and violet dots and line, respectively) and STD_LONG_ of 6 s, and STD relative values from a single saturation (0.75 s; golden points and line). (D) Binding epitope mappings of ESF452 binding to α-glucosidase were obtained by the three different methods.

To validate the efficiency of the rd-STD NMR protocol to significantly reduce the experimental time required for binding epitope mapping determination, we studied the binding of the α-glucosidase enzyme with an sp^2^-iminosugar glycomimetic ligand (ESF45, Figure 1D).

For each proton of the ESF452 ligand, eq 6 was used to obtain the value of STD_0_^app^, using STD_LONG_ as the STD intensity at a 6 s saturation time and STD_SHORT_ at 0.75 s. In Figure 1A and B, we compare the mathematically fitted (following eq 3) STD NMR build-up curves of ESF452 using both a full data set (1A; six data points from 0.5 to 6 s of saturation time) and a reduced data set (1B, 0.75 and 6 s), and the STD_0_ values of each proton were calculated using both approaches.

**Figure 2:**
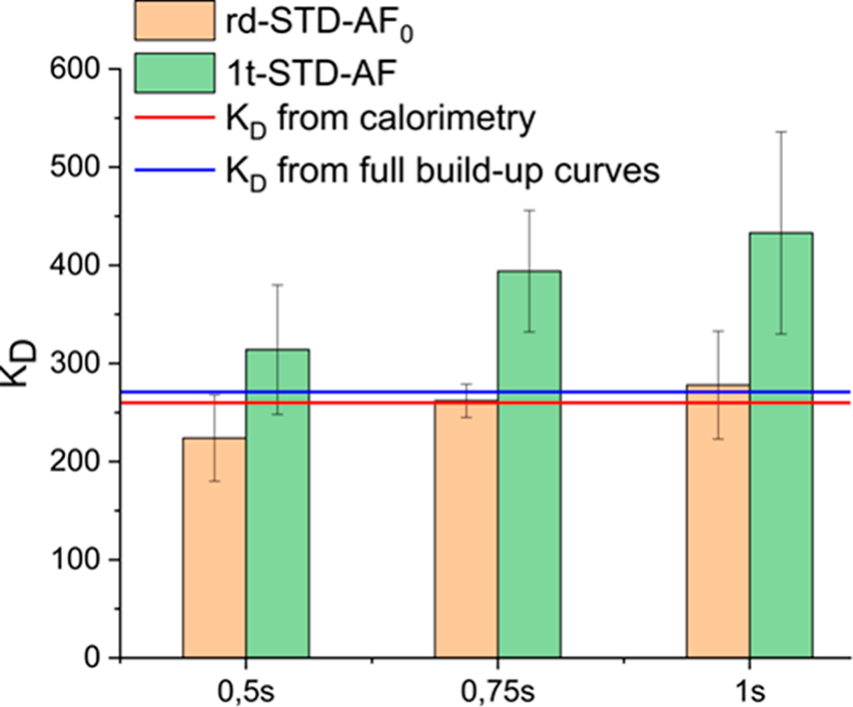
Comparison of the *K*_D_ values obtained using one saturation time only (green bars) and the rd-STD-AF_0_ approach (orange bars) for the RCA120-galactoside complex. The *K*_D_ values obtained by calorimetry^4,15^ and by STD NMR using full build-up curves are shown as red and blue lines, respectively. The *K*_D_ obtained using the rd-STD NMR approach at 0.75 s is almost identical to the values obtained from calorimetry and from full build-up curves.

Figure 1C shows a comparison of the resulting binding epitope mappings of ESF452 using different approximations. The rd-STD NMR approach, using a *t*_SHORT_ of 0.75 s (Figure 1C, blue line; Figure 1D-2), gave almost identical values to those obtained using the traditional full build-up curves (Figure 1C, black line; Figure 1D-1), with an average difference of 1%. Using the rd-STD NMR protocol with a *t*_SHORT_ larger than 1 s can result in STD_SHORT_ values close to the asymptotic value STD_MAX_, leading to very large errors in the determination of *k*_SAT_ and, hence, the STD_0_^app^ values (see eq 6; Figure 1C, violet line). Importantly, the use of a single saturation time (0.75 s) led to a binding epitope mapping deviating much more significantly from the full build-up curve results (Figure 1C, golden line, Figure 1D-3) than using the rd-STD NMR approach with a *t*_SHORT_ of 0.75 s.

To test the performance of the rd-STD NMR approach on different samples, three other α-glucosidase-sp^2^-iminosugar complexes were subjected to the same type of analysis (ligands ESF7, MG277, ONJ, Supporting Information Figures S3, S4, and S5), further validating that the rd-STD NMR approach leads to binding epitope mappings that are almost identical to the much more time demanding full STD build-up curve approximation.

### Validation of the rd-STD NMR Approach 2: Analysis of Apparent Initial Slope Data (STD_0_^app^) from Ligand Titration Experiments. Affinity Determination (*K*_D_) from Binding Isotherms

The great advantage of rd-STD NMR over the traditional approach really comes into play when the latter involves long experimental times, for instance, when determining dissociation constants. For example, while using the traditional build-up curve to construct the protein–ligand binding isotherm can lead to data sets exceeding 30 experiments and 2–3 days of experimental time (e.g., six data points per ligand concentration, and five ligand concentrations), with the rd-STD NMR approach each isotherm data point can be constructed from as few as two experiments, drastically reducing the total experimental time. To further validate the accuracy of the rd-STD NMR approach, we determined the dissociation constant of a well-known system such as the methyl-β-d-galactoside (β-Gal-OMe) bound to agglutinin 120 from *Ricinus communis* (RCA120) and compared it with the value obtained from the analysis of full build-up curves. This sample was chosen because of its robustness, high sensitivity, and high spectral dispersion with respect to other carbohydrates. Previous calorimetric titration studies at 293 K reported a *K*_D_ of 260 μM for the binding of β-Gal-OMe to RCA120.^4,15^

The magnitudes of the apparent dissociation constants determined from the traditional analysis of full build-up curves and the rd-STD NMR approach are shown in Figure 2 (see also Supporting Information Table S1, Table S3, and Figure S8). Using the full curves, a *K*_D_ of 271 μM was obtained, while with the rd-STD NMR approach, using a *t*_SHORT_ of 0.75 s and a STD_LONG_ at 6 s as saturation times, we obtained a value of 262 μM. This result strongly supports that the rd-STD NMR approach is as accurate as the full build-up curve method, yet requiring only a fraction of the experimental time. For our system, the full build-up curves procedure required a total of 44 h of data acquisition, while only 16 h was needed using the novel rd-STD NMR analytical approach, reducing, therefore, the experimental time by more than 60%. Further, we compared those results with a *K*_D_ determined using one single saturation time only at each ligand concentration to construct the binding isotherm. The results showed that a single saturation time approach leads to much higher errors compared to the full curves and the rd-STD NMR approach (Supporting Information Table S2).

To apply the rd-STD NMR approach, STD NMR spectra at two different saturating times must be employed. Following eq 6, one of the data points must be at sufficiently long saturation times to approximate to STD_MAX_ (STD_LONG_, typically 6 or 8 s is a good approximation given typical ranges of *T*_1_ values of small medium molecules),^16^ ensuring the plateau of the build-up curve has been reached. The other data point to consider, STD_SHORT_, should correspond to a saturation time low enough to account for the growth of the STD intensity. The drawback of using very low saturation times (e.g., 0.25–0.5 s) is that it can lead to large errors during integration of STD spectra, for systems producing very weak STD intensities. On the contrary, if *t*_SHORT_ is too large (above 1 s), STD values can become very similar to STD_LONG_, leading to very large errors in the determination of STD_0_^app^. From our experience, we propose using 0.75 s as the best compromise for *t*_SHORT_ (eq 6).

## Conclusions

STD NMR spectroscopy is a uniquely suited ligand-observed NMR technique to characterize weak affinity protein–ligand binding and dissociation constants. However, a major drawback comes from the usually long experimental times required. Herein, we report a new approach, based on a reduced data set analysis using only two STD NMR data points instead of the full STD build-up curve, which reduces the experimental time by more than 60%. Our new rd-STD NMR method allows obtainment of binding epitope mappings and *K*_D_ values that are almost identical to those obtained by the classical full curves approach. We believe rd-STD NMR represents an important step forward in the form of a very powerful approach for academic and industrial settings for fast and accurate characterization of the structural and energetic features governing the formation of weak protein-fragment complexes. We foresee rd-STD NMR making a significant contribution to boost the characterization of target-hit binding features (shorter time required for epitope mapping and *K*_D_ determination), being a key step in fragment-based drug discovery.

## Data Availability

We have developed a Web site, which is freely available, to quickly obtain the dissociation constant using both the Langmuir isotherm and the law of mass action, as well as the values of the ligand epitope mapping. Researchers interested in using the Web site should first contact the corresponding authors to request access to it. The Web site is accessible via the following link https://stdrdweb.streamlit.app/.

## Supporting Information Available

The materials and methods section, which includes the description of the synthesis of compounds, sample preparation, and NMR experiments; ^1^H and ^13^C NMR spectra and binding epitopes of the ligands and the values of dissociation constants; the raw STD NMR data, which includes the integrated intensities of the STD spectra and the fitted parameters of the STD NMR build-up curves (PDF)
