# Supplementary material for: Speeding-up the Determination of Protein–Ligand Affinities by STD NMR: The Reduced Data Set STD NMR Approach (rd-STD NMR)
Source: Anal Chem. 2024 Jan 2;96(2):615–9. doi: 10.1021/acs.analchem.3c03980 (PMC10853903; doi:10.1021/acs.analchem.3c03980)
Supplement: Supplementary file 1 — ac3c03980_si_001.pdf [file ac3c03980_si_001.pdf]

Supporting Information

Speeding-up the Determination of Protein-Ligand Affinities by STD NMR: The Reduced Dataset STD NMR Approach (rd-STD NMR)

Gabriel Rocha¶,[a] Jonathan Ramírez-Cárdenas¶,[a] M. Carmen Padilla-Pérez,[b] Samuel Walpole,[c] Ridvan Nepravishta,[c,d] M. Isabel García-Moreno,[b] Elena M. Sánchez-Fernández,[b] Carmen Ortiz Mellet,[b] Jesús Angulo*,[a, c] Juan C. Muñoz-García*[a]

[a] G. Rocha, J. Ramírez-Cárdenas, Dr J. Angulo, Dr J. C. Muñoz-García
Institute for Chemical Research (IIQ), CSIC – University of Seville
49 Américo Vespucio St, 41092, Seville, Spain
E mail: *j.angulo@iiq.csic.es, *juan.munioz@iiq.csic.es

[b] M. C. Padilla-Pérez, Dr. M. I. García-Moreno, Dr. E. M. Sánchez-Fernández, Prof. C. Ortiz Mellet
Dept. of Organic Chemistry, Faculty of Chemistry, University of Sevilla
Prof. García González 1, 41012, Sevilla, Spain

[c] Dr S. Walpole, Dr R. Nepravishta, Dr J. Angulo
School of Pharmacy, University of East Anglia
Norwich Research Park, NR4 7TJ Norwich, UK

[d] Dr R. Nepravishta
Cancer Research Horizons, The Beatson Institute
Garscube Estate, Switchback Road, Bearsden, Glasgow, G61 1BD, UK

¶ These authors contributed equally to this work

TABLE OF CONTENTS

Materials and methods S2
1. Preparation of sp^2-iminosugars: General methods S2
2. Preparation of sp^2-iminosugars: Synthesis and characterization S2
2.1. Preparation of ESF452 S2
2.2. Preparation of ESF7 S3
3. Sample preparation S4
4. STD NMR experiments S4

Results S5
1. NMR spectra (^{1}H, ^{13}C) S5
2. Binding epitope mappings of ESF7 S8
3. Binding epitope mappings of MG277 S9
4. Binding epitope mappings of ONJ S10
5. Binding epitope mapping of Methyl-β-D-Galactoside S11
6. K_D determination using the Law of Mass Action S11
7. K_D determination using the Langmuir Isotherm S12

Raw STD NMR data S13

REFERENCES S17

Materials and methods

1. Preparation of sp[2]-iminosugars: General methods

All reagents and solvents were purchased from commercial sources and used without further purification. Thin-layer chromatography was performed on precoated TLC plates, silica gel 30F-245, with visualization by UV light and by carrying with 0.2% w/v cerium (IV) sulphate-5% ammonium molybdate in 2 M H_2SO_4. Column chromatography was performed on Chromagel (silice 60 AC.C 70-200 μm and 35-70 μm). Optical rotations were measured at 20 ± 2 ºC in 1 cm tube on a Jasco P-2000 polarimeter using a sodium lamp (λ 589 nm). NMR experiments ^{1}H (^{13}C) were performed at 300 (75.5) and 500 MHz (125.7 MHz) at the NMR Service of the University of Seville Research Technology and Innovation Centre (CITIUS). 2D COSY and HSQC experiments were carried out to assist on NMR assignments. Mass spectra were registered at the Mass Spectrometry Service of the Institute for Chemical Research (unit 824861, IIQ, CSIC-US, Spain) using a Bruker Elute UHPLC system coupled to a Bruker AmaZon SL spectrometer instrument. The system is equipped with an ESI (Electrospray Ionization) ionization source and a Dual Fuel Ion Trap. Samples (2-5 μL of 10-20 μM solutions) were either directly injected or chromatographed using 0.1% formic acid eluting gradients at a flow rate of 0.3 mL/min. Spectra were registered in both positive and negative modes and the results were processed using Bruker Compass HyStar software in the m/z 100-2000 range. All compounds were purified to ≥95% purity as determined by elemental microanalysis results obtained on an elemental analyser Leco CHNS-932 (IIQ, CSIC-US). The analytical results for C, H, N, and S were within ±0.4 of the theoretical values. 3,4-Di-*O*-acetyl-5*N*,6*O*-(oxomethylidene) nojirimycin iminoglycal and (1*R*)-1-*O*-(FmocSer)-2,3,4-tri-*O*-acetyl-5*N*,6*O*-oxomethylidenenojirimycin were prepared according to a previously reported procedure.[1]

2. Preparation of sp[2]-iminosugars: Synthesis and characterisation

The sp[2]-iminosugars **ESF452**, **ESF7**, **ONJ**[2] and **MG277**[3] were synthesised at Prof Carmen Ortiz group, following the procedures described below.

2.1. Preparation of ESF452

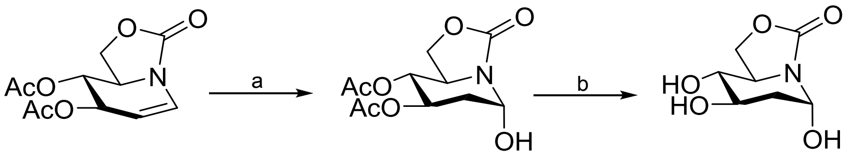

Scheme 1. Reagents and conditions: a) HSePh, CAN, CH_3CN, RT, 24 h, 43%. b) NaOMe (1 M), MeOH, RT, 15 min., quantitative.

(1*R*)-3,4-Di-*O*-acetyl-5*N*,6*O*-oxomethylidene-2-deoxynojirimycin (ESF451). To a stirred solution of di-O-acetyl-iminoglycal[1] (86 mg, 0.34 mmol) and cerium(IV) ammonium nitrate (CAN) (92 mg, 0.17 mmol) in dry acetonitrile (3 mL) under an Ar atmosphere, benzeneselenol (1.70 mmol, 5.0 equiv) was added, and the reaction mixture was stirred for 24h at RT. Et_2O (40 mL) and H_2O (20 mL) were then added, the aqueous phase was extracted with Et_2O (2 × 10 mL), and the organic layer was dried ($MgSO_4$), filtered, and concentrated under reduced pressure. The resulting residue was purified by column chromatography (2:1 EtOAc-cyclohexane). Yield: 40 mg (43%). R_f 0.40 (2:1 EtOAc-cyclohexane). $[\alpha]_D$ +10.9 (c 1.1 in DCM).

^{1}H NMR (500 MHz, $CDCl_3$; Figure S1) δ 5.55 (bs, 1 H, H-1), 5.40 (ddd, 1 H, $J_{2b,3}$ = 11.6 Hz, $J_{3,4}$ = 9.3 Hz, $J_{2a,3}$ = 4.6 Hz, H-3), 4.84 (t, 1 H, $J_{4,5}$ = 9.3 Hz, H-4), 4.39 (t, 1 H, $J_{6a,6b}$ = $J_{5,6a}$ = 8.4 Hz, H-6a), 4.21 (t, 1 H, $J_{5,6b}$ = 8.2 Hz, H-6b), 4.15-4.08 (m, 1 H, H-5), 2.31 (ddd, 1 H, $J_{2a,2b}$ = 13.2 Hz, $J_{2a,1}$ = 2.1 Hz, H-2a), 2.06-2.02 (2 s, 6 H, MeCO), 1.79 (ddd, 1 H, $J_{1,2b}$ = 3.8 Hz, H-2b). ^{13}C NMR (125.7 MHz, $CDCl_3$;

Figure S1) δ 170.5-170.0 (MeCO), 156.0 (CO), 73.7 (C-4), 72.7 (C-1), 68.2 (C-3), 67.1 (C-6), 52.5 (C-5), 35.1 (C-2), 20.9-20.7 (MeCO).

ESIMS: m/z 296.09 $[M + Na]^+$. Anal. Calcd for $C_{11}H_{15}NO_7$: C, 48.35; H, 5.53; N, 5.13. Found: C, 48.28; H, 5.40; N, 4.95.

(1*R*)-5*N*,6*O*-Oxomethylidene-2-deoxynojirimycin (ESF452). Over a stirred solution of (1*R*)-3,4-di-*O*-acetyl-5*N*,6*O*-oxomethylidene-2-deoxynojirimycin (26 mg, 0.09 mmol) in MeOH (2 mL), NaOMe (1 m) (18 mL) was added at RT. The reaction mixture was stirred for 15 min., diluted with MeOH (3 mL), neutralized with solid CO_2 and concentrated under reduced pressure. The resulting residue was purified by column chromatography (9:1 EtOAc-MeOH). Yield: 17 mg (quant.). R_f 0.52 (4:1 EtOAc-MeOH). $[\alpha]_D$ +23.8 (c 0.9 in MeOH).

^{1}H NMR (300 MHz, CD_3OD; Figure S2) δ 5.42 (dd, 1 H, $J_{1,2b}$ = 3.8 Hz, $J_{1,2a}$ = 1.7 Hz, H-1), 4.50 (t, 1 H, $J_{6a,6b}$ = $J_{5,6a}$ = 8.5 Hz, H-6a), 4.22 (dd, 1 H, $J_{5,6b}$ = 6.5 Hz, H-6b), 3.92-3.78 (m, 2 H, H-3, H-5), 3.22 (t, 1 H, $J_{3,4}$ = $J_{4,5}$ = 9.3 Hz, H-4), 2.12 (ddd, 1 H, $J_{2a,2b}$ = 13.3 Hz, $J_{2a,3}$ = 4.6 Hz, H-2a), 1.59 (ddd, 1 H, $J_{2b,3}$ = 11.6 Hz, H-2b). ^{13}C NMR (75.5 MHz, CD_3OD; Figure S2) δ 158.1 (CO), 77.4 (C-4), 74.6 (C-1), 69.5 (C-3), 68.3 (C-6), 55.4 (C-5), 39.4 (C-2).

ESIMS: m/z 234.06 $[M + HCO_2]^-$. Anal. Calcd for $C_7H_{11}NO_5$: C, 44.45; H, 5.86; N, 7.40. Found: C, 44.09; H, 5.53; N, 7.16.

2.2. Preparation of ESF7

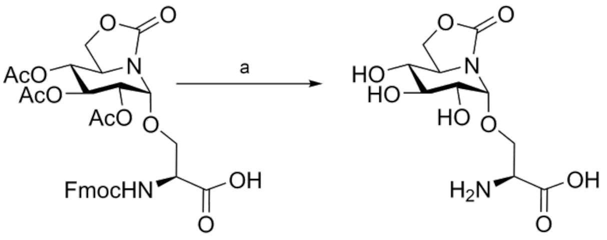

Scheme 2. Reagents and conditions: a) i. 20% Piperidine-MeOH, DCM, RT, 5 h; ii. NaOMe (1M), MeOH, RT, 5 h, 64% in two steps.

(1*R*)-1-*O*-Ser-5*N*,6*O*-oxomethylidenenojirimycin (ESF7). 20% Piperidine-MeOH (180 mL) was added over a stirred solution of (1*R*)-1-*O*-(FmocSer)-2,3,4-tri-*O*-acetyl-5*N*,6*O*-oxomethylidenenojirimycin[1] (120 mg, 0.19 mmol) in DCM (3 mL) and the reaction mixture was stirred at RT for 5 h. The solvent was removed under reduced pressure and the resulting crude filtrated through a pad of SiO_2 (2:1 → 1:2 EtOAc-MeOH). Then, NaOMe (1 M) (111 μL) was added at RT and the reaction was stirred for 5 h, diluted with MeOH (3 mL), neutralized with solid CO_2 and concentrated under reduced pressure. The resulting residue was purified by column chromatography (10:1:1 → 4:1:1 CH_3CN-H_2O-NH_4OH). Yield: 35 mg (64%, global yield). R_f 0.35 (4:1:1 CH_3CN-H_2O-NH_4OH). $[a]_D$ +52.2 (*c* 1.0 in H_2O).

^{1}H NMR (500 MHz, D_2O; Figure S3) δ 5.24 (d, 1 H, $J_{1,2}$ = 4.0 Hz, H-1), 4.74 (t, 1 H, $J_{6a,6b}$ = $J_{5,6a}$ = 9.0 Hz, H-6a), 4.44 (dd, 1 H, $J_{5,6b}$ = 6.0 Hz, H-6b), 4.15 (dd, 1 H, $^2J_{H,H}$ = 10.5 Hz, $^3J_{H,H}$ = 3.0 Hz, OCH_{2Ser}), 4.06 (dd, 1 H, $^3J_{H,H}$ = 7.0 Hz, $CHCO_2H_{Ser}$), 4.00 (ddd, 1 H, $J_{4,5}$ = 10.0 Hz, H-5), 3.90 (dd, 1 H, OCH_{2Ser}), 3.81 (t, 1 H, $J_{2,3}$ = $J_{3,4}$ = 10.0 Hz, H-3), 3.72 (dd, 1 H, H-2), 3.63 (t, 1 H, H-4). ^{13}C NMR (125.7 MHz, D_2O; Figure S3) δ 171.7 (CO_2H), 158.2 (CO), 81.9 (C-1), 73.5 (C-4), 72.4 (C-3), 71.0 (C-2), 67.7 (C-6), 66.5 (OCH_{2Ser}), 54.7 (CH_{Ser}), 53.1 (C-5).

ESIMS: *m/z* 290.9 $[M - H]^-$. Anal. Calcd for $C_{10}H_{16}N_2O_8$: C 41.10, H 5.52, N 9.59. Found: C 40.82, H 5.60, N 9.22.

3. Sample preparation

Five protein-ligand systems were prepared and studied by STD NMR, these are, the RCA120 - β-Gal-OMe complex and those formed by α-glucosidase binding to four different sp^2-iminosugars (**ESF452**, **ESF7**, **MG277** and **ONJ**; Figure 1D, ESI Figures S3D-S6D).
RCA120 was purchased from Vector Laboratories (L-1080-10) and methyl-β-D-galactopyranoside was purchased from Sigma Aldrich (M0285-1G). α-glucosidase was purchased from Sigma Aldrich (G5003-100UN). The sp^2-iminosugars were prepared as described above.
For the binding studies with α-glucosidase and the sp^2-iminosugars, protein and ligand concentrations of 100 μM and 4 mM, respectively, were used. For RCA120 binding to β-Gal-OMe, a series of ligand concentrations (150, 300, 600, 1500, 2500 and 4000 μM) and a total protein concentration of 10 μM were employed. Since RCA120 exists as a tetramer in solution, with one active site per constituent dimer, this is equivalent to a total of 20 μM galactose recognition sites.
All samples of α-glucosidase binding to the four different sp^2-iminosugars were prepared in PBS buffer pH 7.4 in D_2O. RCA120 - β-Gal-OMe sample was prepared in 10 mM phosphate buffer pH 7 in D_2O.

4. STD NMR experiments

STD NMR experiments for α-glucosidase binding to the four different sp^2-iminosugars were carried out at 5 °C using a 600 MHz spectrometer equipped with an UltraShieldPlus superconducting magnet with a high-performance cryoprobe optimised for ^{1}H, ^{13}C, ^{15}N and ^{19}F detection. NMR experiments on the RCA120 - β-Gal-OMe sample were carried out at 298 K using a Bruker Avance III 700 MHz spectrometer.

Results

1. NMR spectra (^{1}H, ^{13}C)

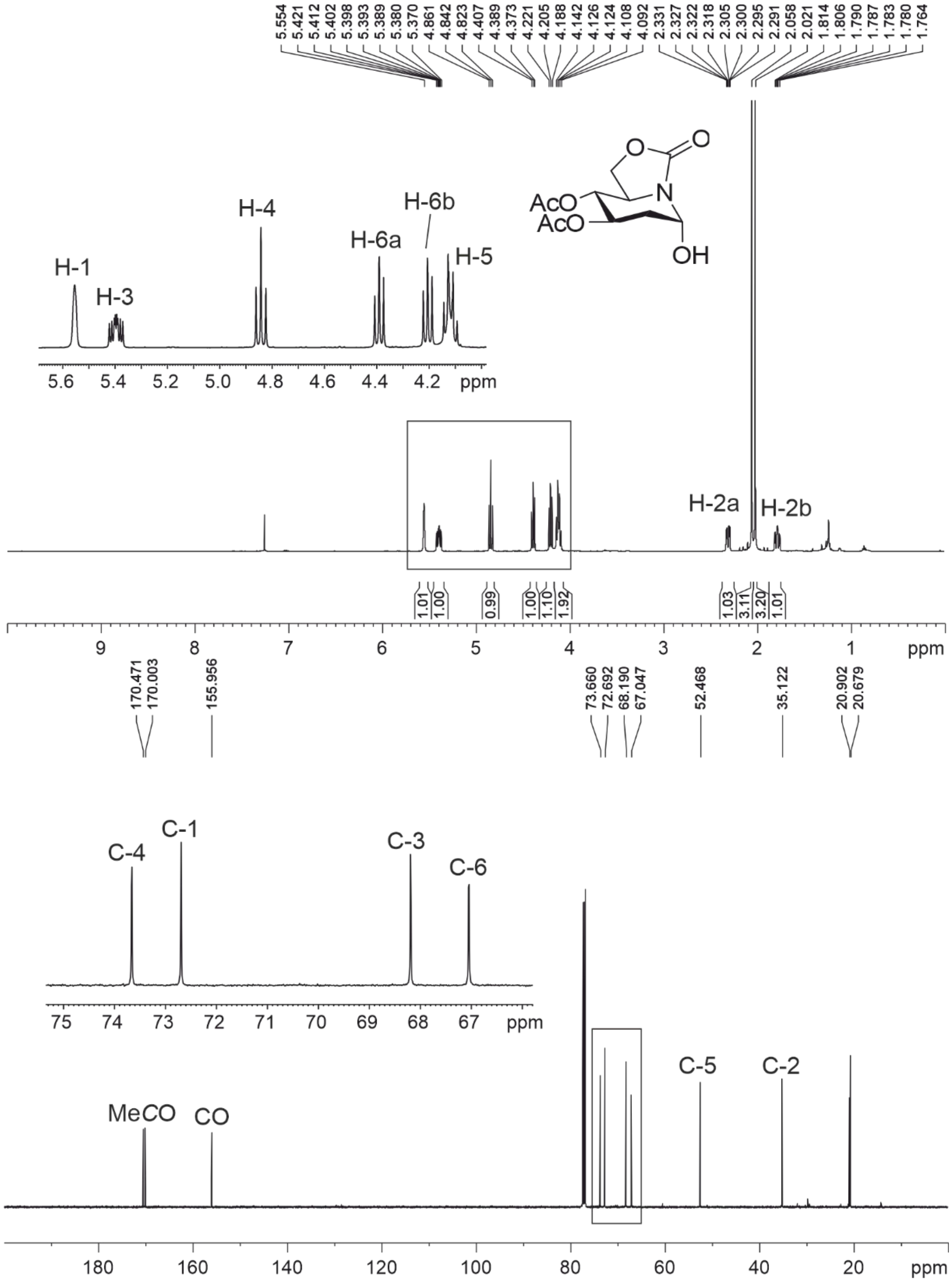

Figure S1. ^{1}H and ^{13}C NMR spectra (500, 125.7 MHz, $CDCl_3$) of **ESF451**.

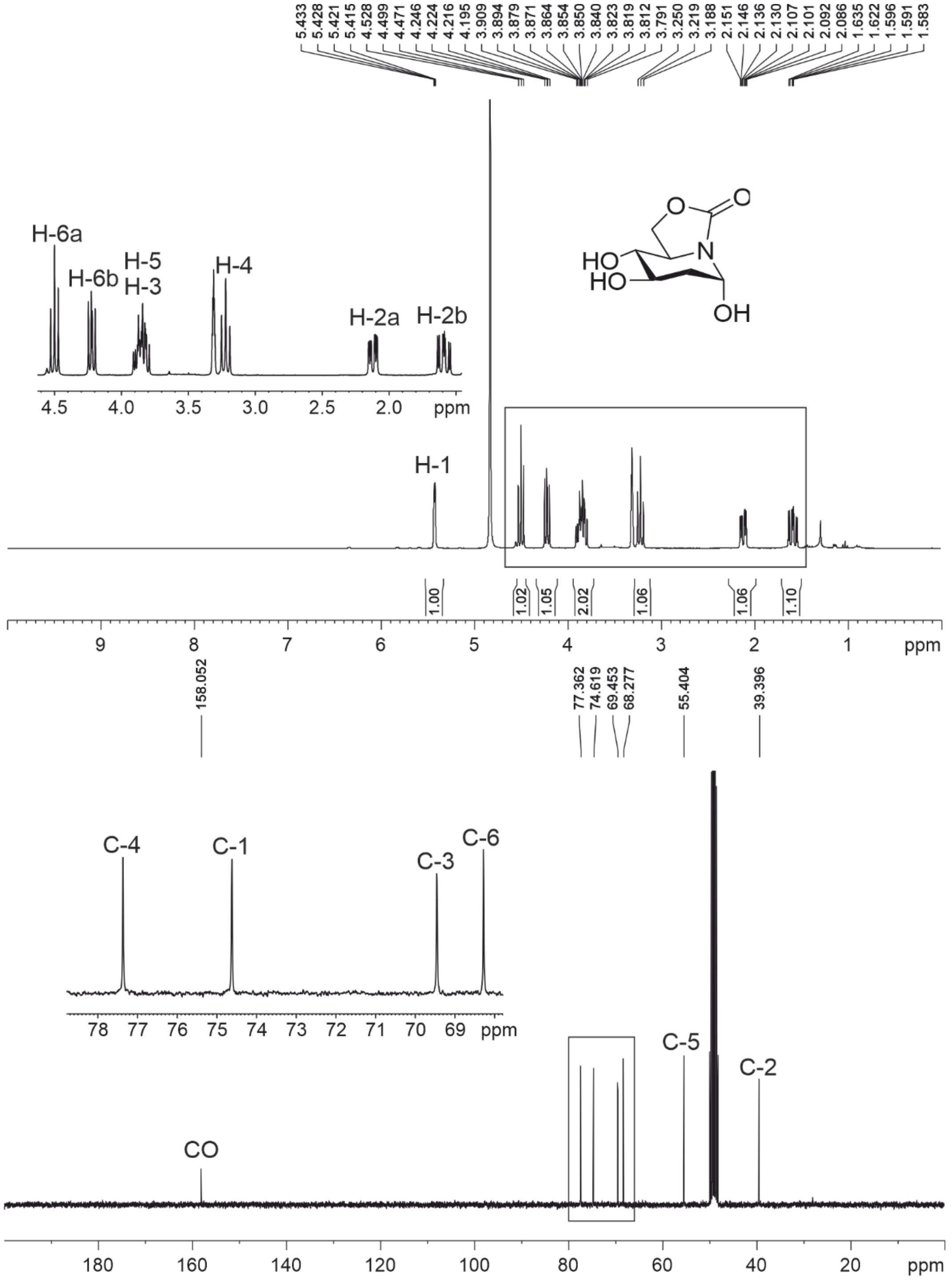

Figure S2. ^{1}H and ^{13}C NMR spectra (300, 75.5 MHz, CD_3OD) of **ESF452**.

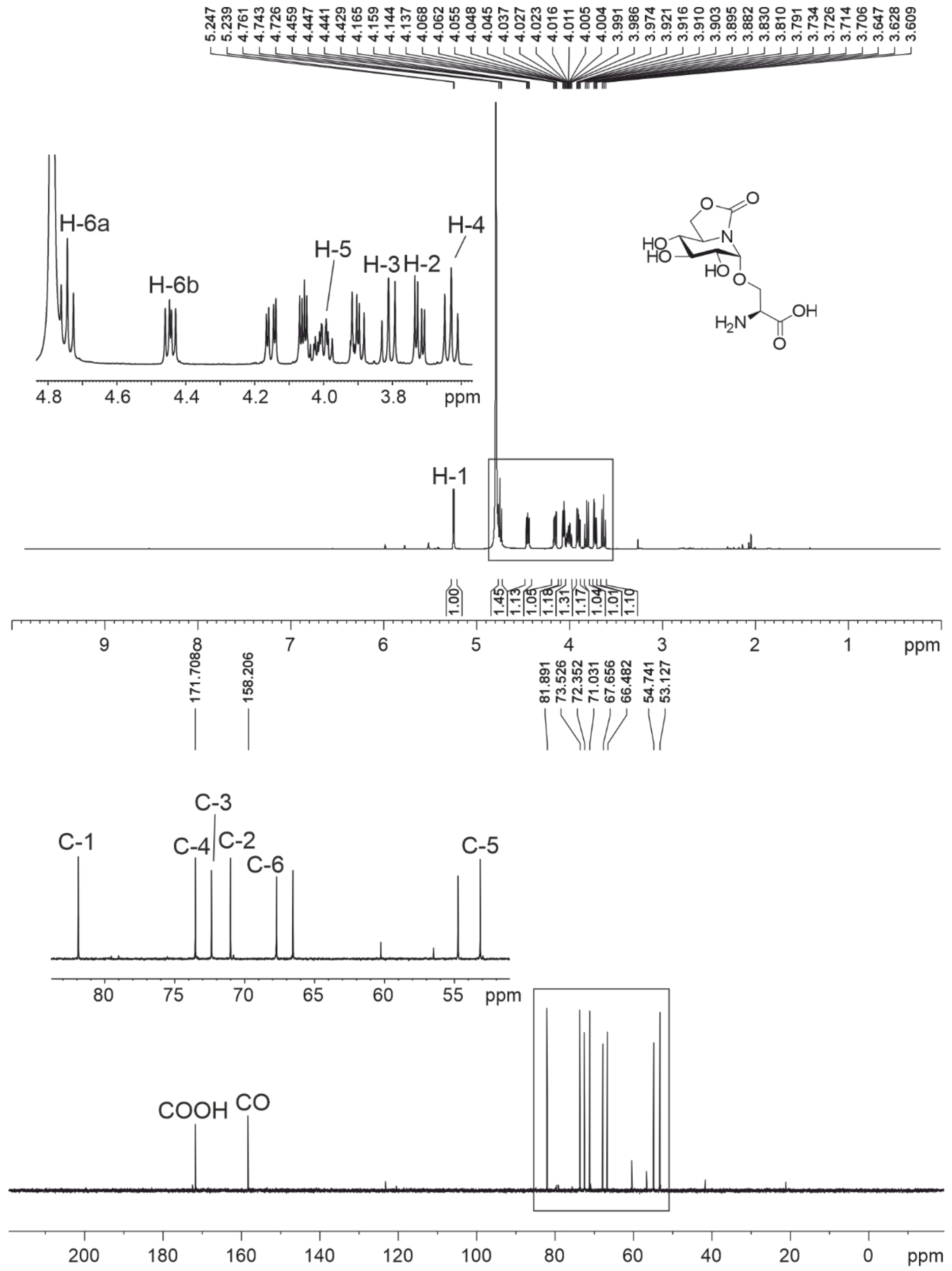

Figure S3. ^{1}H and ^{13}C NMR spectra (500 MHz and 125.7 MHz, D_2O) of **ESF7**.

2. Binding epitope mappings of ESF7

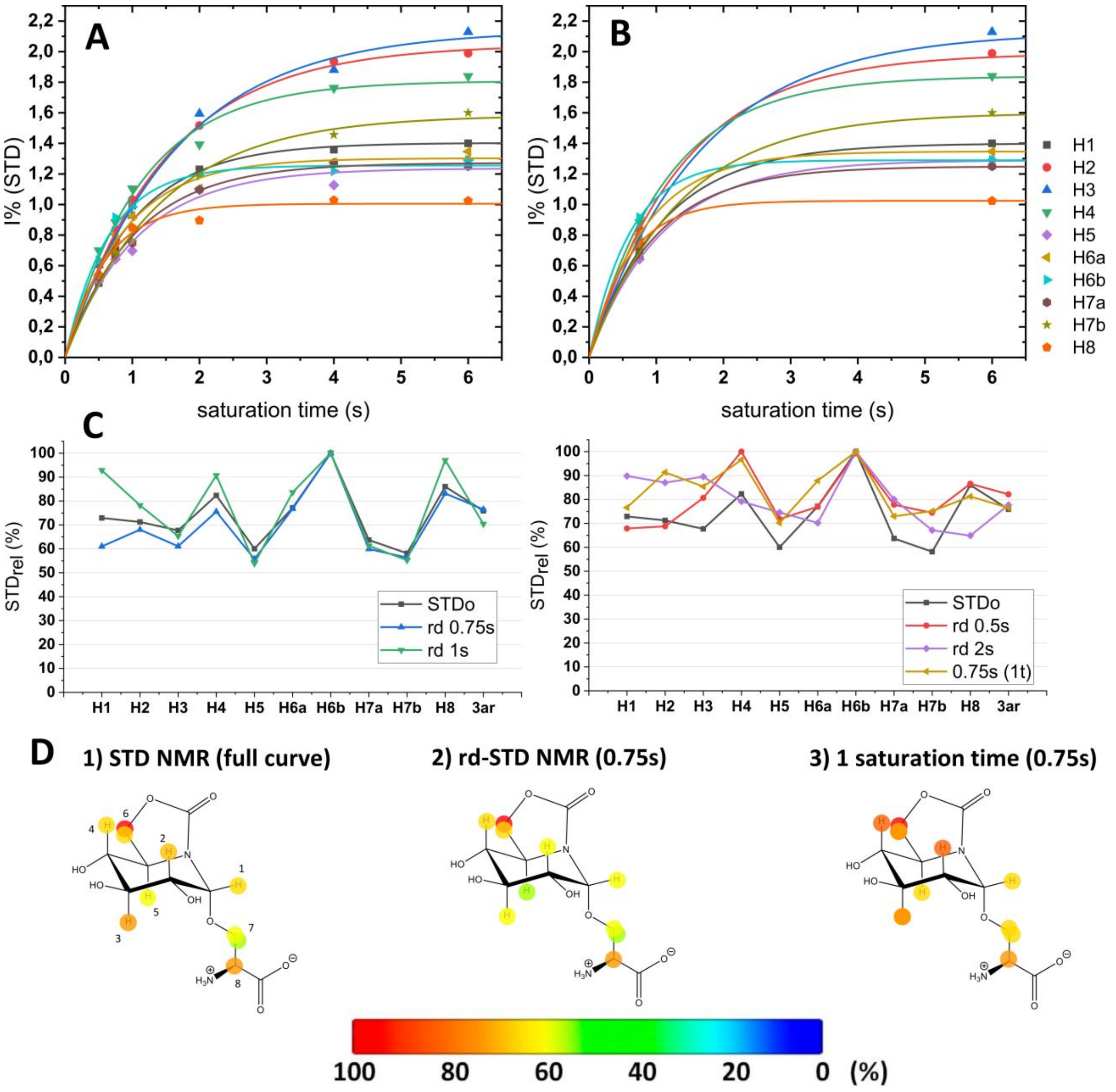

Figure S4. A. STD NMR build-up curve of **ESF7** using all saturation times. **B**. The new reduced dataset approach using STD_{SHORT} at 0.75 and STD_{LONG} at 6 s as experimental data points (right). **C**. Graph showing the STD binding epitope mappings (relative STD values) for each **ESF7** proton obtained using the full build-up curve initial slopes method (black points and line), the new rd-STD NMR approach with t_{SHORT} 0.5, 0.75, 1 and 2 s and t_{LONG} 6 s (red, blue, green and violet points and line), and a single saturation (0.75 s; golden points and line). **D**. Binding epitope mappings obtained by the different methods.

3. Binding epitope mappings of MG277

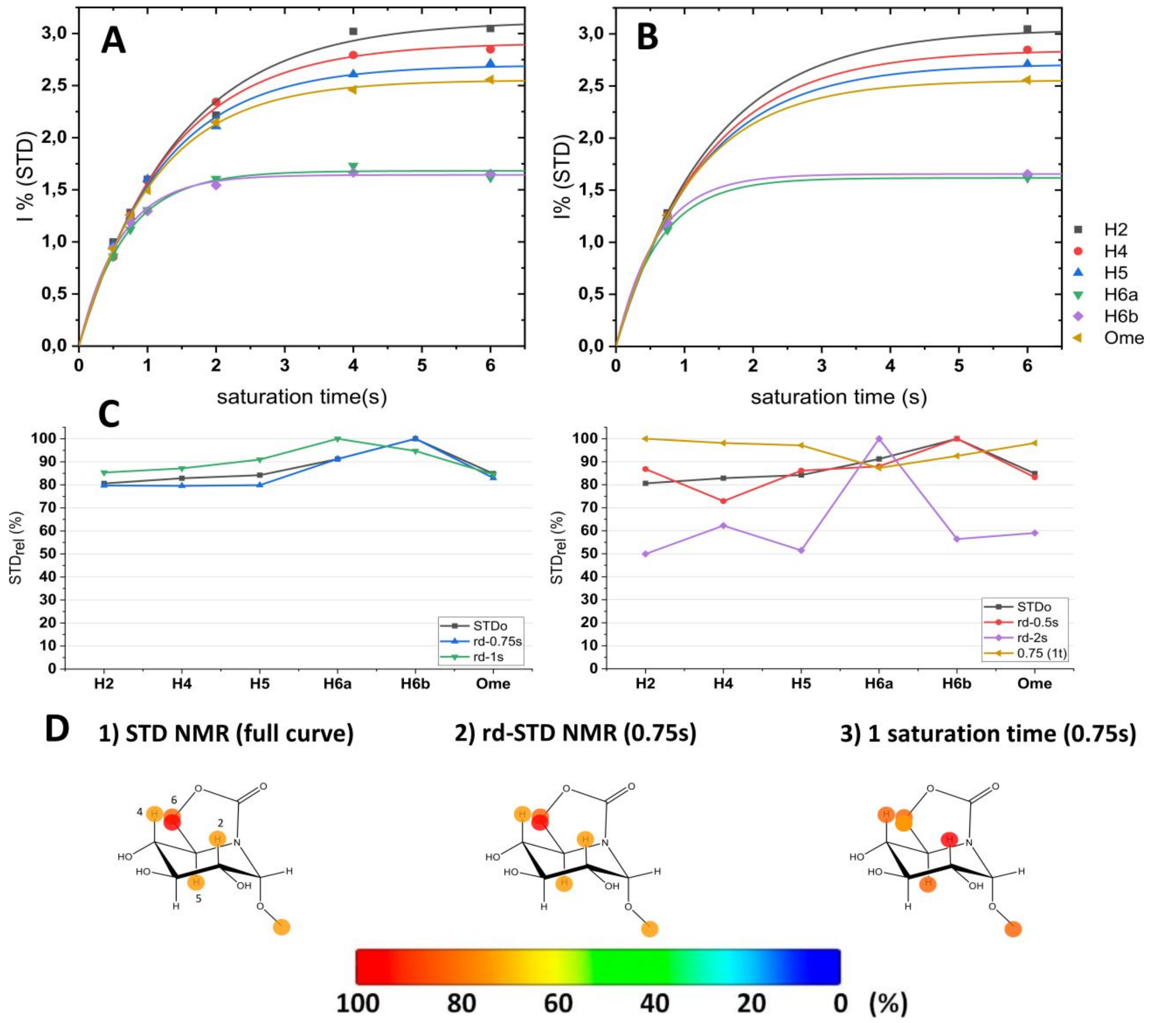

Figure S5. A. STD NMR build-up curve of **MG277** using all saturation times. **B**. The new reduced dataset approach using STD_{SHORT} at 0.75 and STD_{LONG} at 6 s as experimental data points (right). **C**. Graph showing the STD binding epitope mappings (relative STD values) for each **MG277** proton obtained using the full build-up curve initial slopes method (black points and line), the new rd-STD NMR approach with t_{SHORT} 0.5, 0.75, 1 and 2 and t_{LONG} 6 s as STD_{MAX} (red, blue, green and violet points and line), and a single saturation (0.75 s; golden points and line). **D**. Binding epitope mappings obtained by the different methods.

4. Binding epitope mappings of ONJ

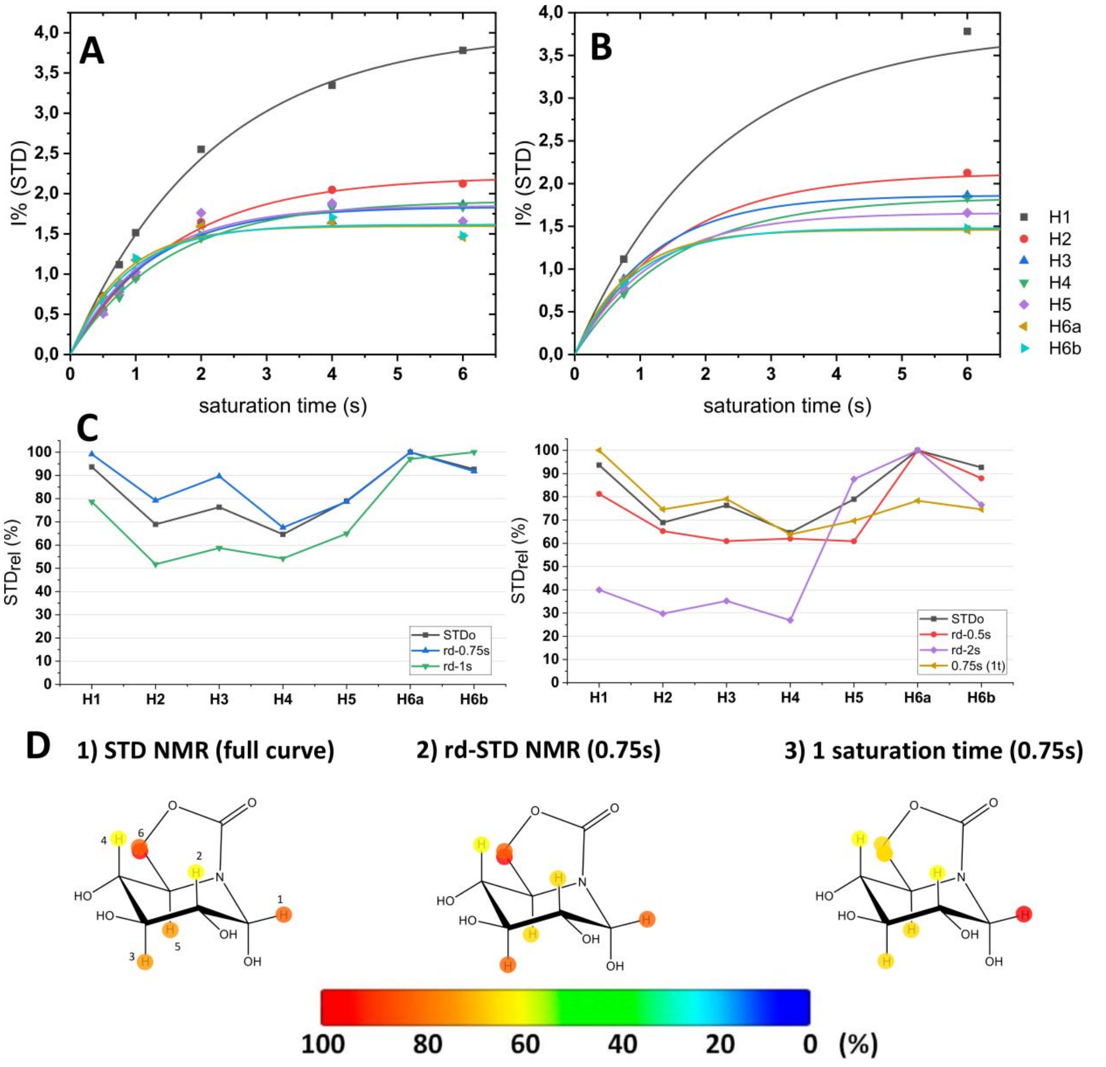

Figure S6. A. STD NMR build-up curve of **ONJ** using all saturation times. **B**. The new reduced dataset approach using STD_{SHORT} at 0.75 and STD_{LONG} at 6 s as experimental data points (right). **C**. Graph showing the STD binding epitope mappings (relative STD values) for each **ONJ** proton obtained from the analysis of the full build-up curves (black dots and line), and the new rd-STD NMR approach with t_{SHORT} 0.5 s (red), 0.75 s (blue), 1 s (green) and 2 s (violet) and t_{LONG} 6 s as STD^{MAX}, and a single saturation time (0.75 s; golden points and line). **D**. Binding epitope mappings obtained by the different methods.

5. Binding epitope mapping of Methyl-β-D-Galactoside

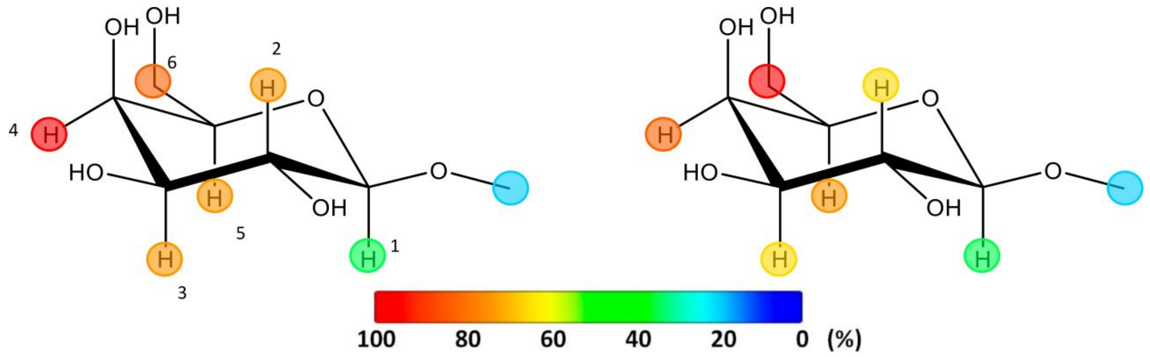

Figure S7. Binding epitope mappings of methyl-β-D-galactoside binding to RCA120 using the full build-up curves (left) and the new rd-STD NMR approach using t_{SHORT} 0.75 s (right).

6. K_D determination using the Law of Mass Action

Table S1. Dissociation constants (μM) calculated for the RCA120-galactoside complex from the Law of Mass Action using the full STD NMR build-up curves (STD-AF_0) and the reduced dataset (rd-STD-AF_0) approximation.

Proton	STD-AF_0	rd-STD-AF_0 (t_{SHORT} 0.5s)	rd-STD-AF_0 (t_{SHORT} 0.75s)	rd-STD-AF_0 (t_{SHORT} 1s)
**H2**	264 ±35	194 ±56	262 ±75	267 ±42
**H3**	249 ±75	179 ±37	240 ±40	233 ±32
**H4**	309 ±92	290 ±62	271 ±12	324 ±33
**H5**	302 ±70	245 ±47	286 ±55	346 ±44
**Ome**	233 ±25	213 ±45	253 ±25	222 ±60
**Average**	271 ±33	224 ±44	262 ±17	278 ±55

Table S2. Dissociation constants (μM) calculated for the RCA120-galactoside complex from the Law of Mass Action using one saturation time only (1t-STD-AF).

Proton	STD-AF (0.5s)	STD-AF (0.75s)	STD-AF (1s)	STD-AF (2s)
**H2**	302 ±72	436 ±92	430 ±33	633 ±139
**H3**	287 ±47	409 ±44	455 ±39	653 ±157
**H4**	419 ±76	440 ±25	557 ±51	750 ±174
**H5**	323 ±51	396 ±62	451 ±46	519 ±88
**OMe**	239 ±47	289 ±56	272 ±56	324 ±39
**Average**	314 ±66	394 ±62	433 ±103	575 ±163

7. K_D determination using the Langmuir Isotherm

In the main text of this manuscript, we report the dissociation constant of the RCA120-galactoside complex calculated from the Law of Action Mas. When performing STD NMR experiments where there is an excess concentration of ligand over the protein, it is reasonable to approximate the concentration of free ligand to the total ligand concentration. Thus, the monitorisation of STD-AF_0 over a series of titrations allows the construction of the association isotherm, which can be fitted to a Langmuir equation[4]:

$$STD - AF_0 = \frac{\alpha_{STD} \cdot [L]}{[L] + K_D}$$

yielding both the equilibrium dissociation constant, K_D, and α_{STD} (a dimensionless scaling factor representing the maximum STD amplification for the monitored signal).

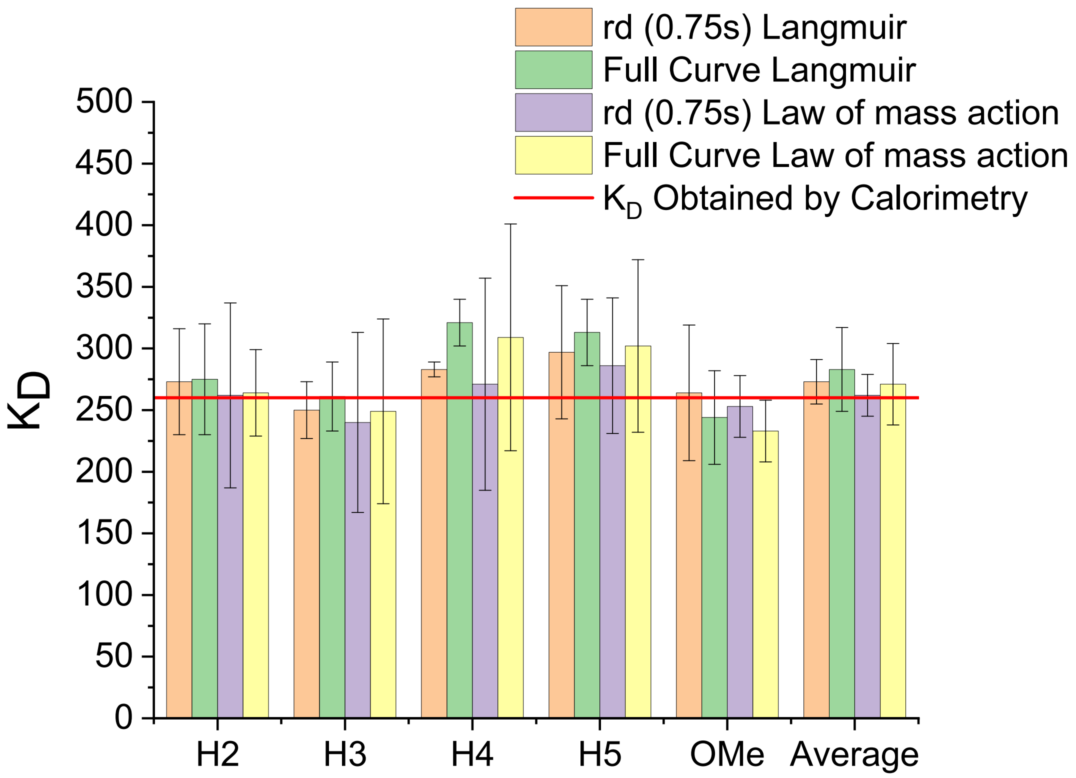

Figure S8. Comparison of the K_D values obtained for the RCA120-galactoside complex for each ligand proton using the Langmuir Isotherm (orange bars – Full Curves method; green bars – rd-STD NMR (t_{SHORT} 0.75 s) approach) and the Law of Mass Action (yellow bars – Full Curves method; violet bars – rd-STD NMR (t_{SHORT} 0.75 s) approach). The K_D value obtained by calorimetry is shown as a red line [5,6].

Table S3. Dissociation constants (μM) from the Langmuir isotherm using the full STD NMR build-up curves (STD-AF_0) and the reduced dataset (rd-STD-AF_0) approximation for the RCA120-galactoside complex.

Proton	STD-AF_0 (Full Curves)	rd-STD-AF_0 (t_{SHORT} 0.5s)	rd-STD-AF_0 (t_{SHORT} 0.75s)	rd-STD-AF_0 (t_{SHORT} 1s)
**H2**	275 ±20	205 ±57	273 ±43	280 ±25
**H3**	261 ±28	190 ±22	250 ±23	244 ±18
**H4**	321 ±21	302 ±35	283 ±6	337 ±18
**H5**	313 ±27	254 ±27	297 ±54	358 ±24
**OMe**	243 ±22	223 ±45	264 ±55	233 ±61
**Average**	283 ± 34	235 ± 44	273 ± 18	290 ± 55

* K_D (calorimetry) [5,6] = 260 μM.

Raw STD NMR data

Table S4. STD intensities (%) at 5 °C of **ESF452** irradiating at 0 ppm.

t_{sat} (s)	0.5	0.75	1	2	4	6
H_1	1.8	2.7	3.4	5.3	6.8	7.0
H_{2eq}	1.8	2.2	2.5	2.9	2.8	2.8
H_{2ax}	2.5	3.1	3.6	4.0	4.0	4.0
H_4	1.4	2.0	2.5	4.0	5.0	5.4
H_{6a}	1.4	1.9	2.3	3.0	3.4	3.4
H_{6b}	1.4	2.0	2.3	3.0	3.2	3.2

Table S5. STD_{MAX}, k_{SAT}, STD_0 and STD_{rel} at 5 °C of **ESF452** irradiating at 0 ppm.

	STD_{MAX}	k_{SAT}	STD_0	STD_{rel}
H_1	7.3	0.63	4.59	57%
H_{2eq}	2.9	1.96	5.61	70%
H_{2ax}	4.0	1.99	8.05	100%
H_4	5.5	0.61	3.39	42%
H_{6a}	3.4	1.10	3.73	46%
H_{6b}	3.3	1.21	3.96	49%

Table S6. STD intensities (%) at 5 °C of **ESF7** irradiating at 0.64 ppm.

t_{sat} (s)	0.5	0.75	1	2	4	6
H_1	0.5	0.7	1.0	1.2	1.4	1.4
H_2	0.5	0.8	1.0	1.5	2.0	2.0
H_3	0.6	0.8	0.9	1.6	1.9	2.1
H_4	0.7	0.9	1.1	1.4	1.8	1.8
H_5	0.5	0.6	0.7	1.1	1.1	1.3
H_{6a}	0.5	0.8	0.9	1.1	1.3	1.3
H_{6b}	0.6	0.9	1.0	1.2	1.2	1.3
H_{7a}	0.5	0.7	0.7	1.1	1.3	1.2
H_{7b}	0.5	0.7	0.8	1.2	1.5	1.6
H_8	0.5	0.7	0.8	0.9	1.0	1.0

Table S7. STD_{MAX}, k_{SAT}, STD_0 and STD_{rel} at 5 °C of **ESF7** irradiating at 0.64 ppm.

	STD_{MAX}	k_{SAT}	STD_0	STD_{rel}
H_1	1.4	1.01	1.42	73%
H_2	2.0	0.68	1.39	71%
H_3	2.1	0.62	1.32	68%
H_4	1.8	0.89	1.61	82%
H_5	1.2	0.95	1.17	60%
H_{6a}	1.3	1.15	1.50	77%
H_{6b}	1.3	1.55	1.95	100%
H_{7a}	1.3	0.98	1.24	64%
H_{7b}	1.6	0.72	1.13	58%
H_8	1.0	1.67	1.68	86%

Table S8. STD intensities (%) at 5 °C of **MG277** irradiating at 0.64 ppm.

t_{sat} (s)	0.5	0.75	1	2	4	6
H_2	1.0	1.3	1.6	2.2	3.0	3.0
H_4	0.9	1.3	1.6	2.3	2.8	2.8
H_5	1.0	1.2	1.6	2.1	2.6	2.7
H_{6a}	0.9	1.1	1.3	1.6	1.7	1.6
H_{6b}	0.9	1.2	1.3	1.5	1.7	1.7
**Ome**	0.9	1.3	1.5	2.1	2.5	2.6

Table S9. STD_{MAX}, k_{SAT}, STD_0 and STD_{rel} at 5 °C of **MG277** irradiating at 0.64 ppm.

	STD_{MAX}	k_{SAT}	STD_0	STD_{rel}
H_2	3.1	0.70	2.2	81%
H_4	2.9	0.77	2.2	83%
H_5	2.7	0.85	2.3	84%
H_{6a}	1.7	1.47	2.5	91%
H_{6b}	1.6	1.65	2.7	100%
**Ome**	2.6	0.90	2.3	85%

Table S10. STD intensities (%) at 5 °C of **ONJ** irradiating at 6.9 ppm.

t_{sat} (s)	0.5	0.75	1	2	4	6
H_1	0.7	1.1	1.5	2.6	3.3	3.8
H_2	0.6	0.8	1.0	1.6	2.0	2.1
H_3	0.5	0.9	1.0	1.6	1.7	1.9
H_4	0.5	0.7	0.9	1.4	1.8	1.8
H_5	0.5	0.8	1.0	1.8	1.9	1.7
H_{6a}	0.7	0.9	1.2	1.6	1.6	1.5
H_{6b}	0.7	0.8	1.2	1.5	1.7	1.5

Table S11. STD_{MAX}, k_{SAT}, STD_0 and STD_{rel} at 5 °C of **ONJ** irradiating at 6.9 ppm.

	**STD_{MAX}**	**K_{SAT}**	**STD_0**	**STD_{rel}**
**H_1**	4.0	0.46	1.87	94%
**H_2**	2.2	0.62	1.37	69%
**H_3**	1.8	0.83	1.52	76%
**H_4**	1.9	0.67	1.29	65%
**H_5**	1.9	0.85	1.57	79%
**H_{6a}**	1.6	1.25	1.99	100%
**H_{6b}**	1.6	1.14	1.85	93%

Table S12. STD intensities (%) at 25 °C of β-Gal-OMe at 150 μM.

**t_{sat} (s)**	**0.5**	**0.75**	**1**	**2**	**4**	**6**
**H_1**	20.3	23.2	24.3	38.6	35.9	30.9
**H_4**	29.5	36.9	40.8	52.5	50.0	55.5
**H_{6a}**	21.1	32.5	36.3	45.5	43.2	38.2
**H_{6b}**	22.6	32.5	32.3	42.5	45.8	44.7
**H_5**	25.6	32.5	34.6	44.2	47.6	47.4
**H_3**	30.2	39.1	45.0	59.2	60.2	59.3
**OMe**	11.2	15.8	19.7	29.1	34.1	35.5
**H_2**	29.7	37.0	38.6	58.7	57.3	58.6

Table S13. STD intensities (%) at 25 °C of β-Gal-OMe at 300 μM.

**t_{sat} (s)**	**0.5**	**0.75**	**1**	**2**	**4**	**6**
**H_1**	10.6	16.8	19.1	25.8	26.3	28.7
**H_4**	22.0	28.8	33.6	42.1	45.9	45.3
**H_{6a}**	16.5	22.1	20.8	30.7	31.9	32.4
**H_{6b}**	14.1	21.7	24.6	31.3	32.5	33.3
**H_5**	17.1	22.1	24.1	32.5	35.9	33.3
**H_3**	20.0	26.3	31.5	42.9	50.2	47.4
**OMe**	7.3	10.5	13.3	20.5	24.9	25.2
**H_2**	20.6	26.6	31.6	43.1	50.9	50.5

Table S14. STD intensities (%) at 25 °C of β-Gal-OMe at 600 μM.

**t_{sat} (s)**	**0.5**	**0.75**	**1**	**2**	**4**	**6**
**H_1**	6.5	10.1	11.3	16.9	21.8	21.2
**H_4**	13.5	19.4	23.0	30.9	34.3	34.2
**H_{6a}**	10.3	14.2	16.4	22.6	23.7	23.3
**H_{6b}**	10.0	16.0	16.4	22.4	25.6	23.5
**H_5**	10.5	14.8	17.1	21.5	23.9	25.4
**H_3**	12.8	18.1	22.7	33.4	39.0	40.2
**OMe**	4.4	6.1	8.0	12.8	16.2	16.6
**H_2**	12.7	16.0	23.6	32.5	38.8	38.8

Table S15. STD intensities (%) at 25°C of β-Gal-OMe at 1500 μM.

t_{sat} (s)	0.5	0.75	1	2	4	6
H_1	2.4	3.9	5.3	9.7	11.1	10.7
H_4	7.4	10.7	12.9	23.2	22.1	22.5
H_{6a}	5.1	7.7	9.1	13.8	13.9	13.1
H_{6b}	5.8	8.2	9.6	14.5	14.3	13.9
H_5	5.7	7.9	9.5	14.3	13.9	13.9
H_3	6.3	9.8	12.2	23.6	25.3	26.2
OMe	2.1	3.3	4.1	7.2	8.8	9.0
H_2	6.0	8.8	12.6	23.0	25.3	26.1

Table S16. STD intensities (%) at 25 °C of β-Gal-OMe at 2500 μM.

t_{sat} (s)	0.5	0.75	1	2	4	6
H_1	1.9	3.1	3.9	5.5	6.9	7.4
H_4	5.0	7.2	9.0	13.5	16.2	16.6
H_{6a}	3.7	5.2	6.6	8.4	9.5	9.4
H_{6b}	3.9	5.5	6.7	8.9	10.0	10.0
H_5	3.8	5.2	6.1	8.7	9.8	9.9
H_3	4.3	6.6	8.3	13.4	18.4	19.6
OMe	1.5	2.2	3.0	4.4	5.9	6.1
H_2	4.6	6.3	8.4	13.5	18.8	19.3

Table S17. STD intensities (%) at 25 °C of β-Gal-OMe at 4000 μM.

t_{sat} (s)	0.5	0.75	1	2	4	6
H_1	1.3	1.7	2.3	3.4	4.7	4.8
H_4	3.6	4.8	6.2	9.7	11.9	12.2
H_{6a}	2.7	3.6	4.3	5.8	6.7	6.7
H_{6b}	2.7	3.8	4.4	6.3	7.3	7.1
H_5	2.5	3.7	4.3	5.9	6.8	7.0
H_3	2.9	4.4	5.6	9.6	13.5	14.4
OMe	1.0	1.5	1.8	3.0	4.0	4.3
H_2	2.9	4.3	5.3	9.2	13.4	14.3

REFERENCES

(1) M. Sánchez Fernández, E.; D. Navo, C.; Martínez-Sáez, N.; Gonçalves-Pereira, R.; J. Somovilla, V.; Avenoza, A.; H. Busto, J.; J. L. Bernardes, G.; Jiménez-Osés, G.; Corzana, F.; M. García Fernández, J.; Ortiz Mellet, C.; M. Peregrina, J. Tn Antigen Mimics Based on Sp2-Iminosugars with Affinity for an Anti-MUC1 Antibody. *Org Lett* **2016**, *18* (15), 3890–3893. https://doi.org/10.1021/acs.orglett.6b01899.

(2) L. Jiménez Blanco, J.; M. Díaz Pérez, V.; Ortiz Mellet, C.; Fuentes, J.; M. García Fernández, J.; C. Díaz Arribas, J.; J. Cañada, F. N-Thiocarbonyl Azasugars: A New Family of Carbohydrate Mimics with Controlled Anomeric Configuration. *Chemical Communications* **1997**, No. 20, 1969–1970. https://doi.org/10.1039/A705755E.

(3) Herrera-González, I.; González-Cuesta, M.; Isabel García-Moreno, M.; Manuel García Fernández, J.; Ortiz Mellet, C. Stereoselective Synthesis of Nojirimycin α-C-Glycosides from a Bicyclic Acyliminium Intermediate: A Convenient Entry to N,C-Biantennary Glycomimetics. *ACS Omega* **2022**, *7* (26), 22394–22405. https://doi.org/10.1021/acsomega.2c01469.

(4) A. Lepre, C.; M. Moore, J.; W. Peng, J. Theory and Applications of NMR-Based Screening in Pharmaceutical Research. *Chem Rev* **2004**, *104* (8), 3641–3676. https://doi.org/10.1021/cr030409h.

(5) Mayer, M.; Meyer, B. Group Epitope Mapping by Saturation Transfer Difference NMR To Identify Segments of a Ligand in Direct Contact with a Protein Receptor. *J Am Chem Soc* **2001**, *123* (25), 6108–6117. https://doi.org/10.1021/ja0100120.

(6) Sharma, S.; Bharadwaj, S.; Surolia, A.; Podder, S. K. Evaluation of the Stoichiometry and Energetics of Carbohydrate Binding to Ricinus Communis Agglutinin: A Calorimetric Study. *Biochemical journal* **1998**, *333* (3), 539–542.
